# Identification and validation of core biomarkers for sepsis: a comprehensive analysis using bioinformatics and machine learning

**DOI:** 10.3389/fimmu.2025.1700704

**Published:** 2025-12-15

**Authors:** Haili Li, Sishi Jiang, Zhibin Chen, Yandong Yao, Muhu Chen, Yingchun Hu

**Affiliations:** Department of Emergency Medicine, The Affiliated Hospital of Southwest Medical University, Luzhou, Sichuan, China

**Keywords:** sepsis, biomarkers, machine learning, immune cells, prognostic assessment, single-cell sequencing, molecular network

## Abstract

Sepsis, a life-threatening condition caused by the body’s response to infection, requires timely and accurate diagnosis to improve patient outcomes. Despite advances in medical research, identifying reliable biomarkers for sepsis remains a challenge. This study aims to identify and validate key biomarkers for sepsis, addressing the limitations of current diagnostic methods like the SOFA score, PCT, and CRP, particularly in terms of specificity and early detection. Methods: We recruited 23 sepsis patients and 10 healthy controls, collecting peripheral blood samples for mRNA sequencing. Public datasets (GSE134347, GSE167363, and GSE220189) were also utilized for differential gene expression analysis. The expression and functions of these biomarkers were systematically verified through GO/KEGG enrichment analysis, protein–protein interaction network construction, ROC curve analysis, AUC values of machine-learning models, survival analysis, and immune cell subset localization analysis. Results: Bioinformatics analysis identified four core biomarkers—CD27, KLRB1, RETN, and CD163—as significantly differentially expressed in sepsis patients. ROC curve and AUC analyses of machine-learning models showed AUC values exceeding 0.9 for these biomarkers across seven models, indicating superior diagnostic performance. Survival analysis revealed significant associations of KLRB1, RETN, and CD163 with sepsis prognosis. Specifically, higher expression levels of RETN and CD163 were linked to increased mortality risk, whereas higher KLRB1 levels were associated with decreased mortality risk. Immune cell-specific expression localization showed CD27 expression in T cells, KLRB1 in NK cells, RETN in monocytes and neutrophils, and CD163 in monocytes, indicating a cell-type-based immune regulatory network. Conclusion: CD27, KLRB1, RETN, and CD163 form a dynamic immune network that reflects the pathological progression of sepsis from hyper-inflammatory to immunosuppressive phases. Monitoring the expression changes of these biomarkers can accurately assess patients’ immune status and guide clinical interventions, such as anti-inflammatory or immunostimulatory therapies. This study offers new directions for early diagnosis and individualized treatment of sepsis.

## Introduction

Sepsis, a life-threatening condition caused by a dysregulated immune response to infection, exhibits distinct pathophysiological features, including an initial hyperimmune response followed by later-stage immunosuppression (1–5). It is one of the leading causes of death among patients in intensive care units (ICUs) globally (6, 7). In 2017, sepsis was responsible for approximately 49 million cases annually worldwide, resulting in approximately 11 million deaths, which constitutes roughly 20% of global mortality. Recent epidemiological studies indicate a decline in both mortality rates and disability-adjusted life years (DALYs) in high-income regions, whereas low-income regions experience the opposite trend, underscoring global disparities in sepsis prevention and treatment (7–9). In China, where economic development is uneven, the cost of treating sepsis imposes a significant financial burden on families in low-income areas and strains the medical insurance system. Therefore, identifying biomarkers with high clinical value is essential for the rapid identification, diagnosis, and treatment of sepsis in these regions. This approach could improve clinical outcomes and alleviate the national economic burden associated with the disease.

In recent years, advancements in molecular biology and immunology have significantly advanced research on sepsis-related biomarkers (10, 11). These studies include both traditional markers such as procalcitonin (PCT) and C-reactive protein (CRP), as well as newer indicators like pre-procalcitonin, miRNA, and molecules found in exosomes (12, 13). These biomarkers are valuable for clinical diagnosis, disease stratification, and monitoring treatment efficacy (14–16). Notably, multimarker combination models, enhanced by machine learning algorithms, have improved diagnostic sensitivity and specificity (17, 18). Despite these advancements, no single biomarker has emerged as a sensitive and specific gold standard for diagnosing sepsis. Traditional microbial culture methods are limited by lengthy processing times and low positive rates, which restrict their use in rapid clinical diagnosis and treatment decisions (19). As a result, biomarkers reflecting the host immune response have become essential tools for auxiliary diagnosis and prognosis assessment.

**Figure 1:**
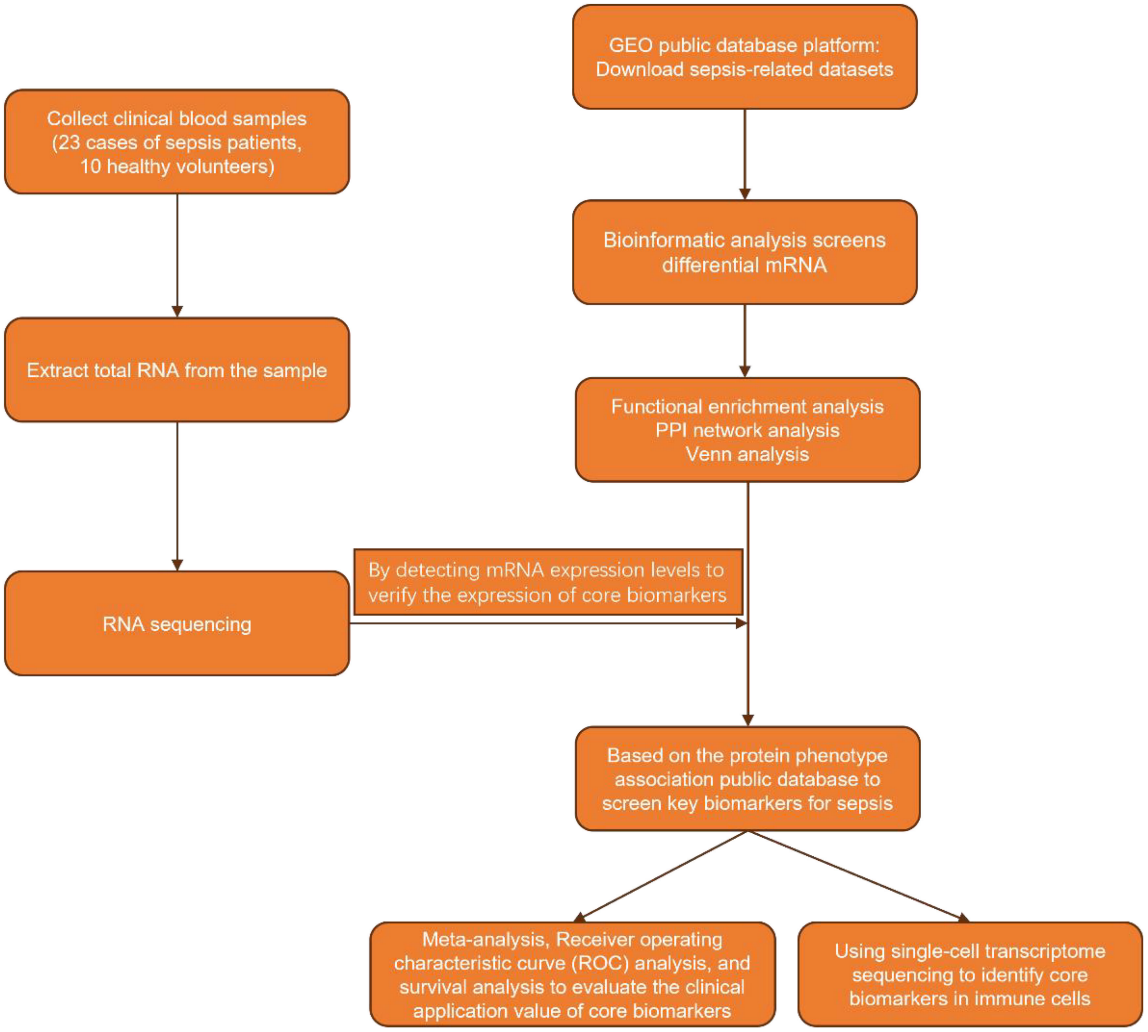
Flowchart of research methods.

Accurate identification of differentially expressed biomarkers is crucial for the early detection of high-risk patients, supporting anti-infection and immunomodulatory treatments, and enabling dynamic monitoring of disease progression. This strategy optimizes treatment plans, ultimately enhancing patient survival rates and quality of life. In our study, we began by identifying differentially expressed mRNAs through bioinformatics analysis. Subsequently, we examined the relationship between the expression levels of proteins encoded by these mRNAs in plasma and the phenotypic manifestations of sepsis, which allowed us to pinpoint key biomarkers. By integrating survival analysis with ROC curve analysis, we assessed the correlation between these biomarkers and the clinical diagnosis and prognosis of sepsis, thereby confirming their clinical utility. Finally, we employed single-cell sequencing to identify the immune cell types associated with core biomarkers, further elucidating their molecular mechanisms (**Figure 1**).

## Materials and methods

### Volunteer recruitment and blood collection

In this study, 23 sepsis patients and 10 healthy controls were recruited from the Emergency ICU of the Affiliated Hospital of Southwest Medical University. Peripheral blood samples were collected for mRNA sequencing to validate the expression levels of core biomarkers identified from public datasets. The dataset generated and analyzed in this study is available in the China National GeneBank Database (CNGBdb) repository at https://db.cngb.org/ with access number CNP0002611. Additionally, data on liver function indicators (alanine aminotransferase, aspartate aminotransferase, direct bilirubin, and total bilirubin), renal function (creatinine, urea, and uric acid), and inflammatory markers (white blood cells, neutrophils, monocytes, and lymphocytes) were collected from the study participants. An unpaired t-test was used for statistical analysis of these indicators. The revised inclusion criteria for sepsis cases are 1) meeting the diagnostic criteria for sepsis; 2) no history of acute or chronic liver disease; 3) no history of severe infectious diseases or immunodeficiency; 4) first-diagnosed cases; and 5) no history of metabolic diseases. Exclusion criteria include 1) major organ dysfunction; 2) concomitant malignancies; 3) immunosuppressive therapy in the past 3 months; and 4) severe congenital diseases or deformities. All participants signed informed consent forms. The research protocol was reviewed and approved by the Ethics Committee of the Affiliated Hospital of Southwest Medical University (ethics number: ky2018029). The clinical trial registration number is ChiCTR1900021261.

### RNA sequencing

Total RNA was extracted from blood samples using the TRIzol method (Invitrogen, Carlsbad, California, USA). Quantification was conducted with an Agilent 2100 Bioanalyzer (Thermo Fisher Scientific, Massachusetts, USA). Initially, specific oligonucleotides were designed to target particular sequences, and ribosomal RNA (rRNA) was removed using RNase H reagent. The RNA was then fragmented at high temperatures in the presence of divalent cations and purified with SPRI beads. Subsequently, the RNA fragments were reverse-transcribed into first-strand cDNA using reverse transcriptase and random primers. Second-strand cDNA was synthesized with DNA polymerase I and RNase H. The library’s quality and concentration were evaluated by analyzing fragment size distribution with an Agilent 2100 Bioanalyzer and quantifying the library using real-time quantitative PCR (QPCR) with TaqMan probes. The sequencing library underwent paired-end sequencing on the BGISEQ-500/MGISEQ-2000 platform at BGI in Shenzhen, China. Finally, mRNA sequencing data were filtered using SOAPnuke (https://github.com/BGI-flexlab/SOAPnuke), and the clean reads were stored in FASTQ format (20).

### Screening of differentially expressed mRNAs

To identify differentially expressed mRNAs in sepsis, we accessed three datasets—GSE134347 (21), GSE167363 (22), and GSE220189 (23)—from the GEO database. GSE134347 comprises transcriptome sequencing data from 156 sepsis patients and 83 healthy individuals. In contrast, GSE167363 and GSE220189 are single-cell RNA sequencing datasets, including samples from 10 and 21 sepsis patients, and 2 and 23 healthy individuals, respectively. For our analysis, we randomly selected data from 50 sepsis patients and 50 healthy individuals from GSE134347. After normalization and quality control, we employed principal component analysis (PCA) to identify and exclude outlier samples. We then conducted differential expression analysis, identifying genes with |log2FC|≥1.5 and a false discovery rate (FDR) < 0.05 as significantly differentially expressed. These analyses utilized the iDEP0.96 (24) online tool and the Xiantao Academic online tool. For a combined analysis, we randomly selected five sepsis patients and two healthy individuals from the GSE167363 dataset, along with five healthy individuals from the GSE220189 dataset. Initially, we assessed the quality of the original single-cell sequencing data. Using Cell Ranger software, we performed data preprocessing and gene expression quantification. After removing low-quality cells, we normalized gene expression and conducted quantitative analysis of cell composition and gene expression. Finally, we performed differential expression analysis on the processed data, selecting genes with |log2FC|≥0.58 and FDR < 0.05 as significantly differentially expressed.

### Gene Ontology and Kyoto Encyclopedia of Genes and Genomes pathway analysis

Gene Ontology (GO) and Kyoto Encyclopedia of Genes and Genomes (KEGG) enrichment analyses are essential bioinformatics tools used extensively to evaluate gene or protein function (25–27). These methods systematically assess the roles of genes in biological processes, cellular components, and molecular functions. By performing detailed statistical analyses on large gene datasets, enrichment analysis identifies functional categories or pathways that are significantly enriched under specific biological conditions. This process enhances our understanding of complex biological mechanisms, providing crucial insights and a solid foundation for further research. In this study, we conducted a comprehensive GO/KEGG enrichment analysis on previously identified differentially expressed mRNAs. The goal was to explore these mRNAs’ molecular functions in detail, thereby identifying biomarkers with higher diagnostic accuracy. We utilized the online tool CNSknowall for GO/KEGG enrichment analysis and visualization.

### Protein interaction analysis

The STRING database (https://string-db.org/) is an essential tool for accessing known protein interactions and predicting new ones. It leverages the genomic context of encoding genes to deduce functional associations among proteins. As a precomputed global resource, STRING facilitates the exploration and analysis of these associations. The protein–protein interaction (PPI) network visually depicts the relationships between proteins, which are crucial for biological functions (28). To identify commonalities between two omics datasets, we employed Venn diagram analysis to pinpoint intersecting genes, which then informed the construction of a PPI network. For our analysis, we established a minimum confidence score of 0.4 to ensure the reliability of the interaction network.

### Identification and validation of core genes

We assessed the expression levels of core mRNAs within the network through peripheral blood RNA sequencing data. Initially, we normalized the raw data. We then conducted an independent samples t-test to determine intergroup differences in gene expression, setting a significance threshold of p < 0.05. For visualization, we employed the “CNSKnowall” online analysis platform to generate box plots.

Subsequently, we analyzed the association between proteins corresponding to mRNAs with significant intergroup differences and sepsis clinical endpoints. This analysis utilized the Atlas of the Plasma Proteome in Health and Disease online database (https://proteome-phenome-atlas.com/), a public repository detailing associations between approximately 3,000 plasma proteins and around 1,000 health-related phenotypes and genomes from approximately 50,000 UK Biobank adults. The disease data were last updated in November 2023 (29). The median follow-up period for all participants was 14.8 years, and results were summarized in a three-line table.

### ROC curve analysis based on machine learning

This study used the GSE134347 dataset to train and evaluate seven widely used machine-learning classifiers. We assessed the predictive performance of identified core biomarkers and plotted receiver operating characteristic (ROC) curves to quantify model discrimination. Machine learning handles complex, non-linear relationships without strong distributional assumptions, enabling discovery of latent patterns in multivariate, real-world data. Its adaptive algorithms automatically tune parameters to accommodate varying data characteristics, allowing more effective performance across diverse tasks and environments than many traditional methods. Combined with scalable computation, this flexibility and adaptability make machine learning a powerful approach for large-scale data analysis and prediction. The seven classifiers applied were K-Nearest Neighbors, Random Forest, Support Vector Machine, Gaussian Naive Bayes, Logistic Regression, Decision Tree, and eXtreme Gradient Boosting (XGBoost) (30).

### Survival analysis

In this study, we performed a survival analysis of core biomarkers by utilizing public databases to explore the link between core biomarker expression and sepsis prognosis. We sourced the GSE65682 dataset from the GEO database, which includes peripheral blood transcriptome sequencing data and detailed clinical follow-up information for 479 sepsis patients (31). After downloading the dataset, we normalized the original expression profile data. For the survival analysis, we used GraphPad Prism 10.0.3 to generate Kaplan–Meier curves and assessed group differences with the log-rank test, considering a significance level of P < 0.05.

### Immune cell localization of core genes

To analyze core gene expression patterns in cell subpopulations using single-cell transcriptome data, the following procedure is employed: First, the Harmony algorithm is utilized to eliminate batch effects, achieving optimal cell clustering through SNN clustering. Next, UMAP dimensionality reduction is applied to visualize the cell community structure. The presto algorithm is then used to identify differentially expressed genes, specifically those with logFC > 0 and min. pct > 0.25, in each cell population. Cell types are annotated using the SingleR database (32). The final quantitative analysis reveals significant differential expression of core genes in specific immune cell types.

## Results

### Clinical information

The study involved 23 sepsis patients and 10 healthy controls. The sepsis group included 15 men and 8 women, whereas the control group had an equal gender distribution of 5 men and 5 women. In the sepsis group, 10 patients died within 28 days, whereas 13 survived; no deaths were reported in the control group during the observation period. Clinically relevant indicators are expressed as mean ± standard deviation, with detailed data available in **Table 1**.

**Table 1:** Clinically relevant information of the sample.

Item	Normal(n=10)	Sepsis(n=23)	P_Value
Gender(M/F)	5/5	15/8	–
28-DayFinale(S/D)	13/10	10/0	–
Age(years)	53.5 ± 7.663	56.7 ± 17.35	0.5826
Leukocytes (10^9^/L).	6.877 ± 1.848	13.05 ± 7.051	0.0109
Neutrophils (10^9^/L).	4.128 ± 1.151	13.74 ± 12.97	0.0272
Monocytes (10^9^/L).	0.443 ± 0.1832	0.8161 ± 1.06	0.2813
lymphocytes (10^9^/L).	2.02 ± 0.5712	1.12 ± 1.533	0.0835
Total bilirubin	16.79 ± 6.368	31.38 ± 37.85	0.2387
Direct bilirubin (µmol/L).	5.24 ± 2.048	16.27 ± 14.62	0.0251
Indirect bilirubin (µmol/L).	11.55 ± 4.362	10.89 ± 10.53	0.8499
ALT(u/L)	20.94 ± 6.443	86.71 ± 179	0.2584
AST(u/L)	22.18 ± 4.526	142.5 ± 272.2	0.176
Creatinine (µmol/L)	63.75 ± 9.259	121.4 ± 128.5	0.1697
Urea (mmol/L)	5.046 ± 1.489	12.8 ± 13.85	0.09
Uric acid (µmol/L)	371.4 ± 62.72	309.2 ± 196.8	0.3395

### Screening of differentially expressed mRNAs

**Figure 2:**
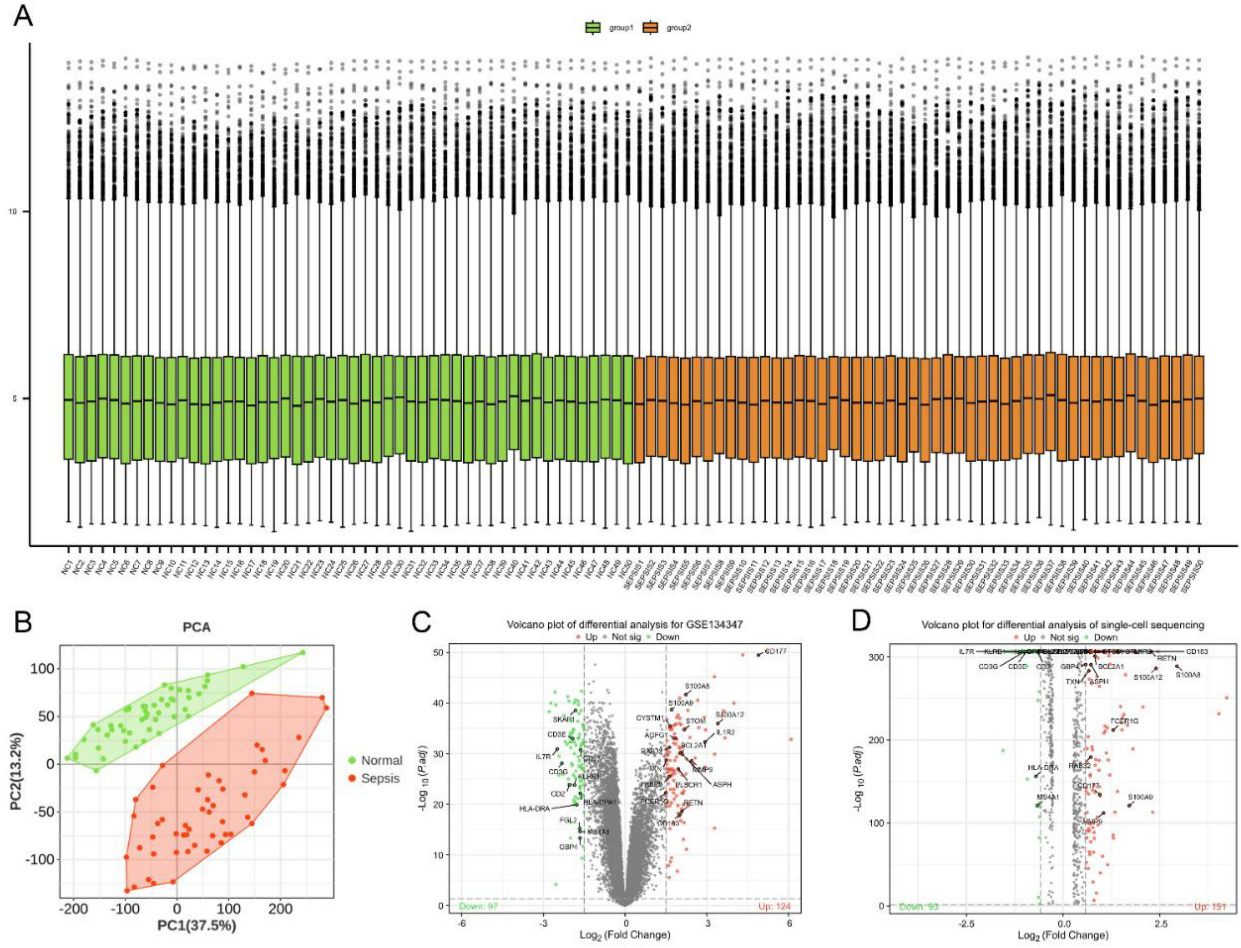
Screen for sepsis-related differentially expressed genes. **(A)** Boxplot. The x-axis represents samples, and the y-axis represents the specific values of genes. The points above and below the box in the boxplot represent gene outliers. The green color indicates the normal healthy group, and the orange color indicates the sepsis patient group. **(B)** PCA plot, also known as principal component analysis. Principal component 1 is on the x-axis, and principal component 2 is on the y-axis. Each point represents a sample. **(C, D)** Volcano plots show differentially expressed genes. The fold change is shown on the x-axis, and the negative logarithm of the P-value is shown on the y-axis. Green indicates downregulated and differentially expressed genes, whereas red indicates upregulated and differentially expressed genes.

In this study, we performed standardization and quality control analysis on the GSE134347 dataset. The findings demonstrated a uniform distribution and strong intra-group consistency, highlighting significant transcriptomic differences between the sepsis and healthy control groups (**Figures 2A, B**). Differential analysis revealed 221 differentially expressed mRNAs, with 124 upregulated and 97 downregulated in the sepsis group (**Figure 2C**). Similarly, analysis of the GSE167363 and GSE220189 datasets identified 244 differentially expressed mRNAs, with 151 upregulated and 93 downregulated in sepsis (**Figure 2D**).

### GO and KEGG pathway analyses

**Figure 3:**
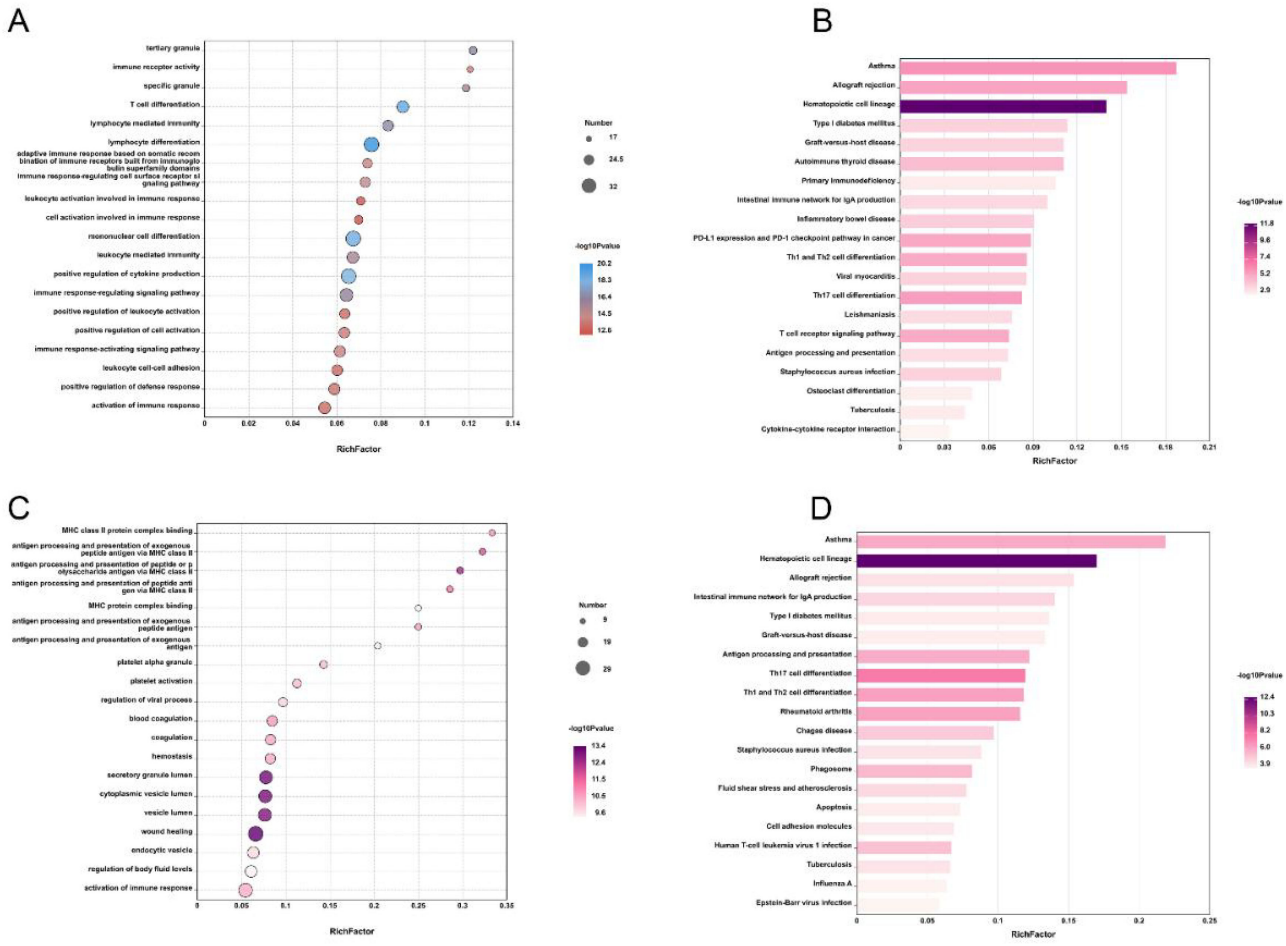
GO and KEGG enrichment analyses of differentially expressed genes. Bubble plots **(A)** and bar charts **(B)** of GO and KEGG enrichment analysis of differentially expressed genes in the GSE134347 dataset, where the horizontal axis represents the enrichment fold and the vertical axis represents the enrichment categories. Bubble plots **(C)** and bar charts **(D)** of GO and KEGG enrichment analysis of the GSE167363 and GSE220189 datasets, where the horizontal axis represents the enrichment fold and the vertical axis represents the enrichment categories.

The GO enrichment analysis of the GSE134347 dataset identified that the differentially expressed mRNAs are predominantly linked to functions such as tertiary granules, immune receptor activity, and specific granules. These mRNAs are also involved in signaling pathways related to T-cell differentiation, monocyte differentiation, leukocyte-mediated immunity, and immune response regulation (**Figure 3A**). The KEGG pathway enrichment analysis revealed that these mRNAs are enriched in disease-related pathways, including asthma, allograft rejection, hematopoietic cell lineage, type 1 diabetes, graft-versus-host disease (GVHD), and autoimmune thyroid disease. Furthermore, they are connected to immune signaling pathways like Th1 and Th2 cell differentiation, T-cell receptor signaling, antigen processing and presentation, Staphylococcus aureus infection, and cytokine–cytokine receptor interaction (**Figure 3B**). In the GO enrichment analysis of single-cell transcriptome data, the differentially expressed mRNAs are primarily associated with MHC class II protein complex binding, antigen processing and presentation, blood coagulation, secretory granule functions, and immune response activation (**Figure 3C**). The KEGG pathway enrichment analysis further demonstrated their involvement in pathways such as asthma, the immune network related to intestinal IgA production, antigen processing and presentation, rheumatoid arthritis (RA), Staphylococcus aureus infection, apoptosis, and viral infection (**Figure 3D**). These findings indicate that the differentially expressed mRNAs are significantly enriched in various immune-related functions and signaling pathways, providing crucial insights into the mechanisms of sepsis.

### Analysis of protein–protein interactions

**Figure 4:**
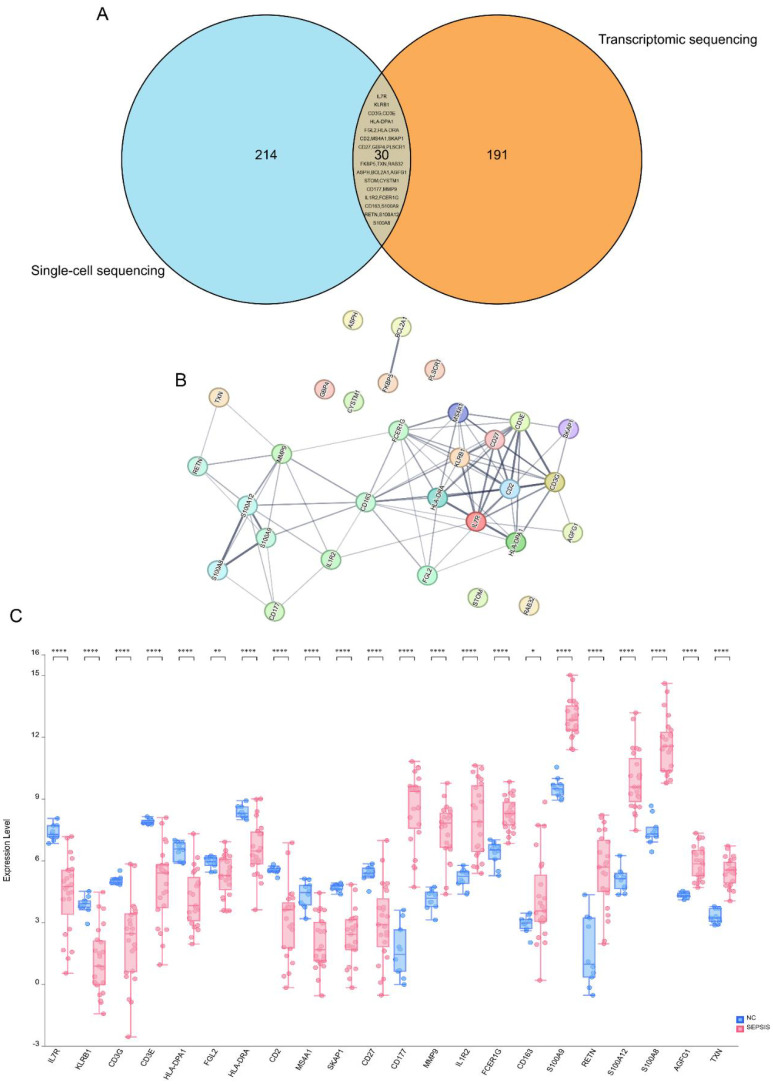
Screening of core biomarkers. **(A)** Venn diagram. Blue represents the differentially expressed genes in the GSE167363 and GSE220189 datasets, and orange represents the differentially expressed genes in the GSE134347 dataset. The Venn diagram shows that there are 30 overlapping genes among the three groups of datasets. **(B)** PPI network diagram. In this study, the protein–protein interaction (PPI) network of the core differentially expressed genes in sepsis was analyzed. The results showed that genes such as CD3E, HLA-DPA1, FGL2, HLA-DRA, CD2, MS4A1, SKAP1, CD27, CD177, MMP9, IL1R2, FCER1G, CD163, S100A9, RETN, and S100A12 are at the core nodes of the PPI network. **(C)** Box plots of differences among multiple groups. Blue represents the healthy normal group, and red represents the sepsis patient group. *p < 0.05; **p < 0.01; ***p < 0.001; ****p < 0.0001; ns (no significance), p > 0.05.

Building on our previous analysis, we performed a Venn analysis that identified 30 differentially expressed mRNAs common to both the GSE134347 dataset and the single-cell dataset (**Figure 4A**). Following this, we constructed a PPI network, highlighting mRNAs such as IL7R, KLRB1, CD3G, CD3E, HLA-DPA1, FGL2, HLA-DRA, CD2, MS4A1, SKAP1, CD27, CD177, MMP9, IL1R2, FCER1G, CD163, S100A9, RETN, S100A12, S100A8, AGFG1, and TXN as central components (**Figure 4B**). These mRNAs show significant potential as core biomarkers. Additionally, we validated their expression levels using clinical samples, confirming that they matched the levels observed in the public dataset (**Figure 4C**).

### Correlation analysis between core biomarkers and sepsis

After the initial screening, we analyzed the disease association of 21 potential sepsis biomarkers. The odds ratio (OR) analysis identified CD27 as having the highest odds ratio, highlighting its strong association with clinically overt sepsis and suggesting its critical role in the disease’s onset. KLRB1 and RETN also showed a strong positive correlation, whereas HLA-DRA, CD163, and IL7R were significantly upregulated. In contrast, proteins such as SKAP1 and IL1R2 exhibited either a protective trend or no significant association. Other proteins, including CD3E, CD3G, MMP9, and S100A12, showed varying degrees of association, although some lacked statistical significance (**Table 2**). The hazard ratio analysis reinforced CD27’s highest risk association, further linking it to clinically overt sepsis. KLRB1, RETN, CD163, and FGL1 significantly increased the risk, whereas MMP9 and TXN also showed notable risk elevation. Interestingly, IL7R demonstrated a protective effect, suggesting a potential role in disease defense. Proteins such as CD3E, CD3G, and SKAP1 did not exhibit a significant risk association, whereas CD2 and CD177 were linked to an increased risk. This analysis underscores the importance of multiple immunomodulatory proteins, particularly CD27, KLRB1, RETN, and CD163, in the risk of clinically overt sepsis, suggesting their potential as candidates for disease prognosis and treatment targets (**Table 3**).

**Table 2:** Association analysis between core candidate proteins and sepsis (OR value).

Disease	Protein	Protein definition	NB individual	NB case	OR[95%CI]	P value
Explicit sepsis	CD163	Scavenger receptor cysteine-rich type 1 protein M130	45586	174	2.04 [1.54-2.70]	5.79E−07
Explicit sepsis	CD27	CD27 antigen	45536	174	4.48 [3.58-5.62]	7.72E−39
Explicit sepsis	CD3E	T-cell surface glycoprotein CD3 epsilon chain	38893	145	1.33 [0.97-1.82]	7.22E−02
Explicit sepsis	CD3G	T-cell surface glycoprotein CD3 gamma chain	38485	140	0.83 [0.65-1.04]	1.11E−01
Explicit sepsis	HLA-DRA	HLA class II histocompatibility antigen, DR alpha chain	45204	173	2.47 [1.80-3.40]	2.27E−08
Explicit sepsis	IL1R2	Interleukin-1 receptor type 2	45295	173	0.57 [0.30-1.09]	9.08E−02
Explicit sepsis	IL7R	Interleukin-7 receptor subunit alpha	45404	174	1.61 [1.24-2.10]	3.77E−04
Explicit sepsis	KLRB1	Killer cell lectin-like receptor subfamily B member 1	45904	173	3.54 [2.59-4.83]	1.89E−15
Explicit sepsis	MMP9	Matrix metalloproteinase-9	45331	174	1.10 [0.88-1.37]	3.87E−01
Explicit sepsis	RETN	Resistin	45294	174	2.50 [2.02-3.10]	4.47E−17
Explicit sepsis	S100A12	Protein S100-A12	44746	173	1.24 [1.04-1.48]	1.59E−02
Explicit sepsis	SKAP1	Src kinase-associated phosphoprotein 1	44967	171	0.73 [0.60-0.89]	1.70E−03

**Table 3:** Risk ratio analysis of the association between core candidate proteins and sepsis risk (HR value).

Disease	Protein	Protein definition	NB individual	NB case	HR[95%CI]	P value
Explicit sepsis	CD163	Scavenger receptor cysteine-rich type 1 protein M130	47362	1950	1.65 [1.51-1.80]	2.60E−29
Explicit sepsis	CD177	CD177 antigen	47097	1942	1.05 [1.02-1.09]	2.47E−03
Explicit sepsis	CD2	T-cell surface antigen CD2	39755	1551	1.18 [1.06-1.31]	2.97E−03
Explicit sepsis	CD27	CD27 antigen	47316	1954	2.55 [2.35-2.76]	3.75E−119
Explicit sepsis	CD3E	T-cell surface glycoprotein CD3 epsilon chain	40336	1588	0.93 [0.81-1.08]	3.46E−01
Explicit sepsis	CD3G	T-cell surface glycoprotein CD3 gamma chain	39906	1561	1.02 [0.97-1.08]	4.50E−01
Explicit sepsis	FGL1	Fibrinogen-like protein 1	41115	1623	1.46 [1.35-1.57]	3.86E−24
Explicit sepsis	HLA-DRA	HLA class II histocompatibility antigen, DR alpha chain	46973	1942	1.20 [1.08-1.34]	8.61E−04
Explicit sepsis	IL1R2	Interleukin-1 receptor type 2	47058	1936	1.22 [1.00-1.49]	5.39E−02
Explicit sepsis	IL7R	Interleukin-7 receptor subunit alpha	47174	1944	0.86 [0.80-0.93]	1.38E−04
Explicit sepsis	KLRB1	Killer cell lectin-like receptor subfamily B member 1	47701	1970	1.95 [1.77-2.16]	1.27E−40
Explicit sepsis	MMP9	Matrix metalloproteinase-9	47099	1942	1.29 [1.21-1.38]	9.22E−14
Explicit sepsis	RETN	Resistin	47045	1925	1.65 [1.52-1.80]	2.28E−32
Explicit sepsis	S100A12	Protein S100-A12	46491	1918	1.14 [1.08-1.20]	1.68E−06
Explicit sepsis	SKAP1	Src kinase-associated phosphoprotein 1	46724	1928	0.98 [0.93-1.04]	5.13E−01
Explicit sepsis	TXN	Thioredoxin	40445	1594	1.17 [1.05-1.29]	3.07E−03

### ROC curve analysis and survival analysis

**Figure 5:**
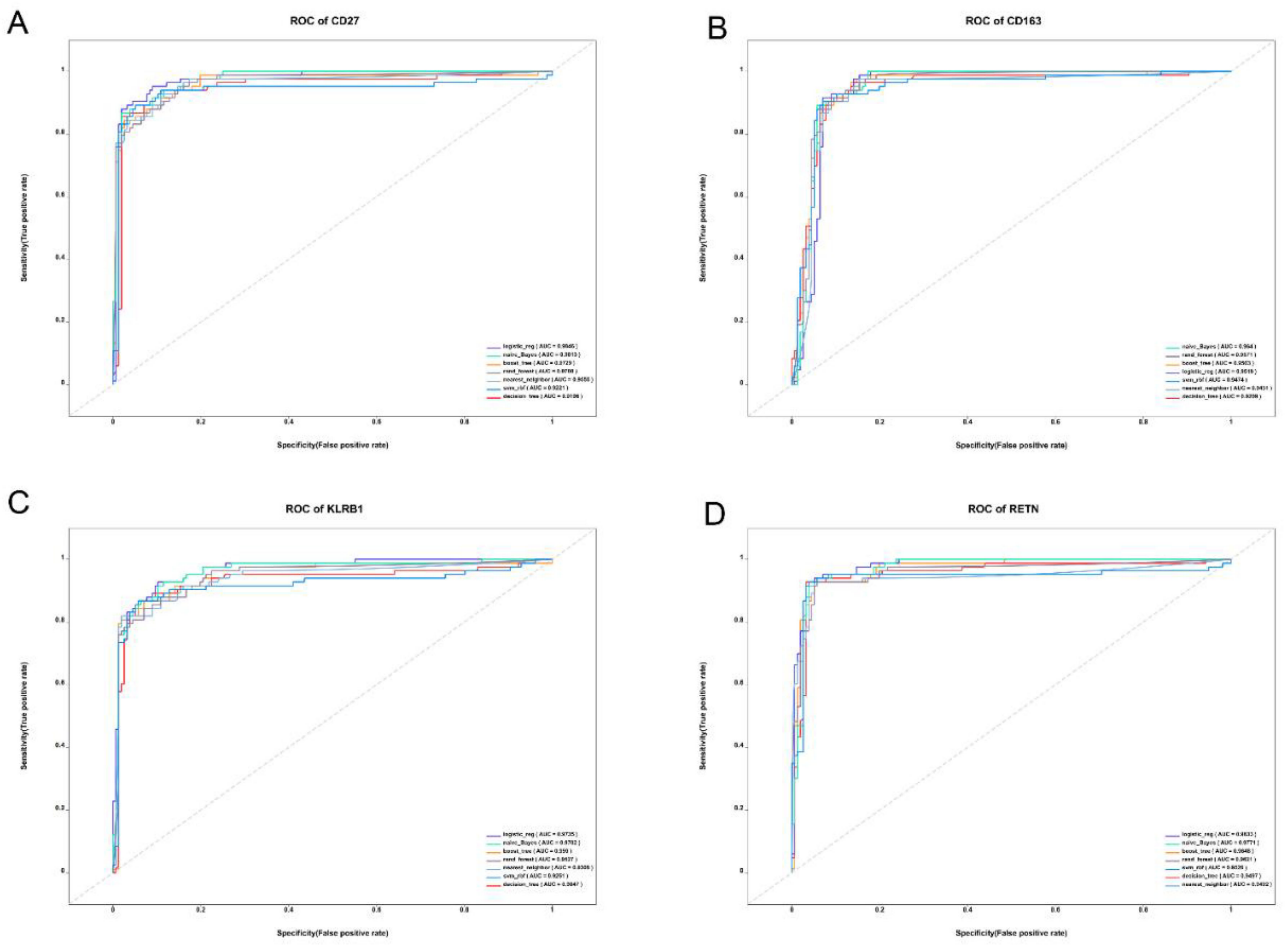
ROC curves of core genes. **(A–D)** ROC curves based on GSE134347 from the GEO database. Specificity is represented on the horizontal axis, whereas sensitivity is represented on the vertical axis. The results show that CD27, KLRB1, RETN, and CD163 exhibit high levels of sensitivity and specificity, with all AUC values greater than 0.9.

**Figure 6:**
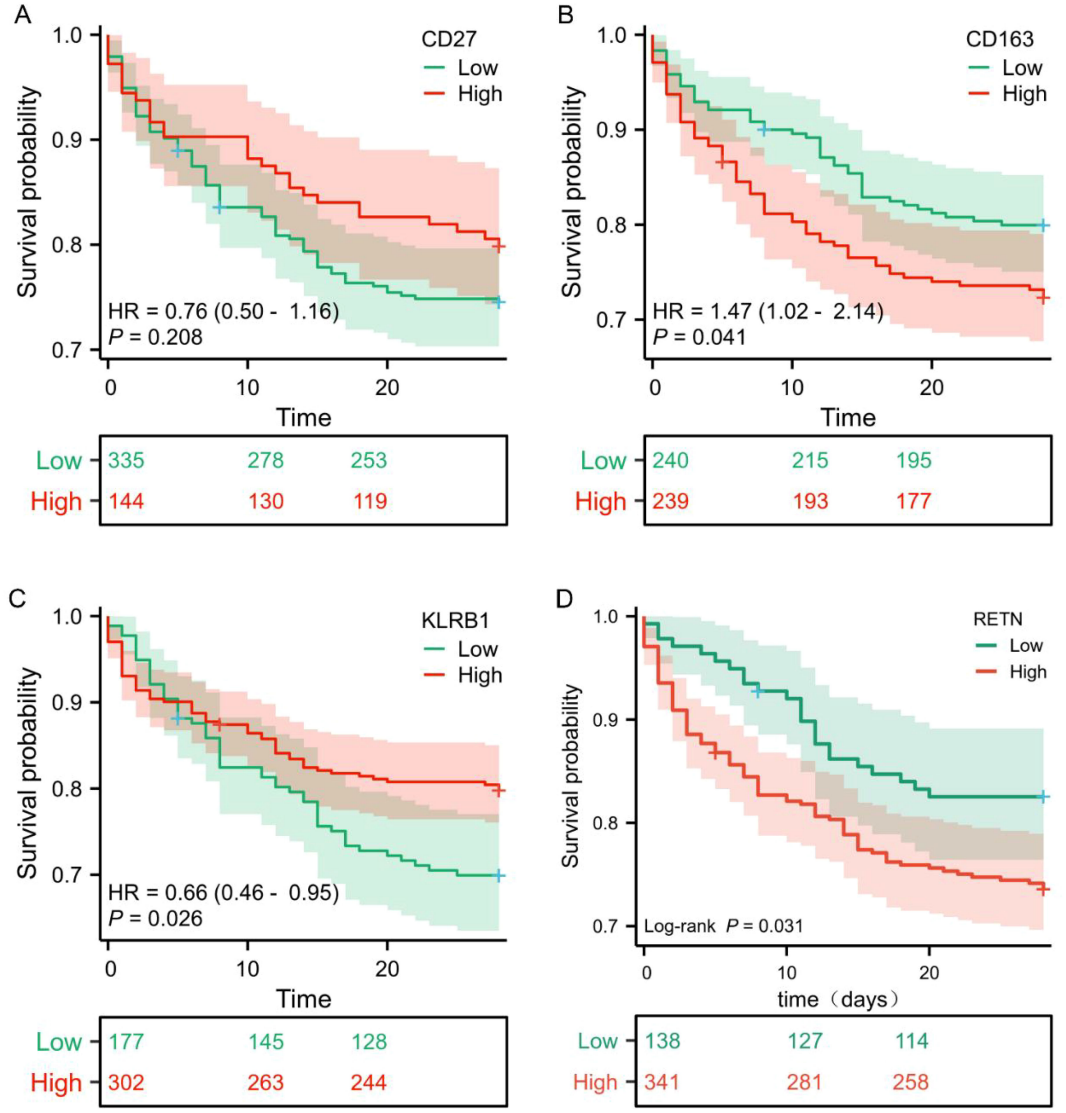
Survival curves of core genes. **(A–D)** Survival curves of core genes plotted based on the GEO database GSE65682. The red line represents the high-expression group, and the green line represents the low-expression group. The survival rate is on the vertical axis, and the horizontal axis shows the 28-day survival period. Compared with patients in the high-expression group, patients with low expression of KLRB1 had a lower 28-day survival rate, indicating that the expression level of KLRB1 was positively correlated with the survival rate of sepsis patients (P < 0.05). In addition, patients in the low-expression groups of CD163 and RETN had a higher 28-day survival rate than those in the high-expression groups, and their expression levels were negatively correlated with the survival rate of sepsis patients (P < 0.05). There was no statistically significant correlation between the expression level of CD27 and the survival rate.

In this study, we applied seven widely used machine learning methods to classify and train samples, evaluating the predictive performance of CD27, KLRB1, RETN, and CD163 using ROC curve analysis. The results showed that the AUC values for these biomarkers exceeded 0.9 across all models (**Figures 5A–D**), indicating their strong potential as sepsis biomarkers. Further survival analysis revealed that while the expression levels of KLRB1, RETN, and CD163 significantly influenced sepsis prognosis, CD27 did not demonstrate statistical significance (**Figures 6A–D**). Interestingly, RETN and CD163 were positively correlated with the risk of sepsis-related death, whereas KLRB1 was negatively correlated with this risk.

### Immune cell localization of core biomarkers

**Figure 7:**
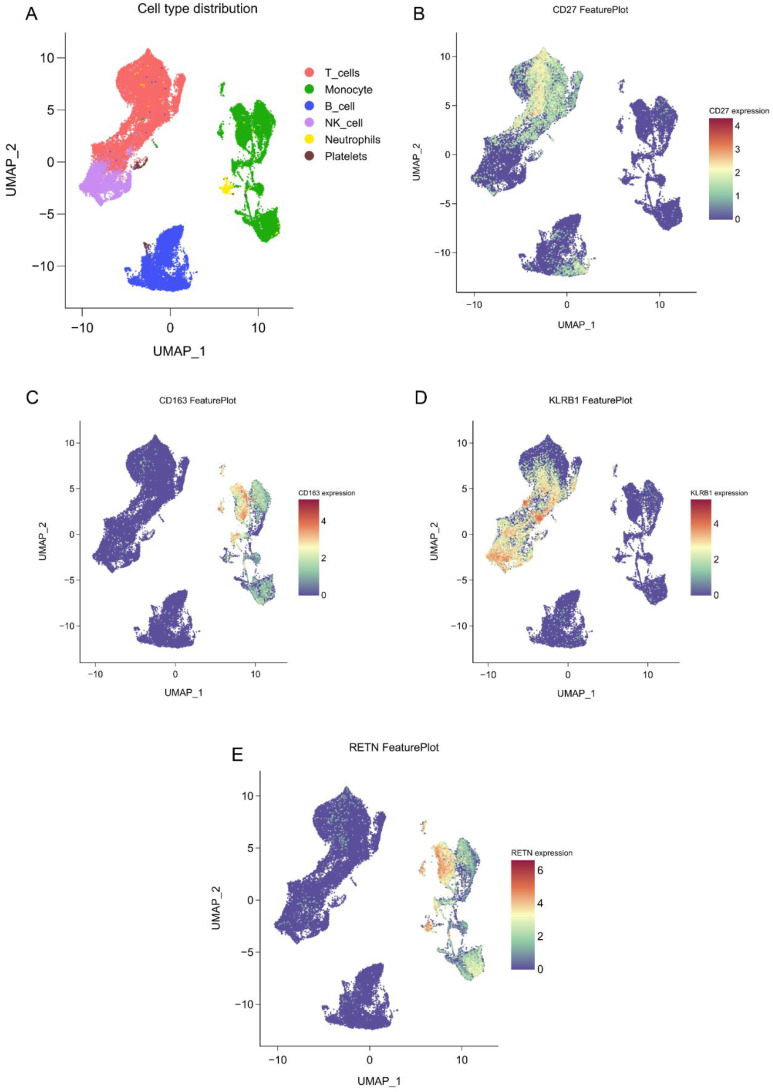
Single-cell spatial atlas of core genes. **(A)** Two-dimensional UMAP plot after PCA dimensionality reduction, where each small dot represents a unit. Red indicates T cells, green indicates monocytes/macrophages, blue indicates B cells, purple indicates NK cells, yellow indicates neutrophils, and brown indicates platelets. **(B-E)**. Expression distribution of CD27, KLRB1, RETN, and CD163 in human blood PBMCs.

Single-cell sequencing data analysis indicates that CD27 is predominantly expressed on T cells, CD163 is mainly found on monocytes, and RETN is present in both monocytes and neutrophils. This distribution suggests that RETN may play dual roles in metabolism and inflammatory responses. Furthermore, KLRB1 is highly expressed on natural killer cells (**Figures 7A–D**). These findings provide a foundation for further exploration of the roles various immune cells play in disease progression. Future research should concentrate on the functions and mechanisms of these biomarkers within specific disease contexts, particularly their interactions with other immune cells, to reveal more complex immune networks.

## Discussion

We identified and validated CD27, KLRB1, RETN, and CD163 as key biomarkers of sepsis by integrating transcriptome and single-cell data using bioinformatics and machine learning. These molecules exhibit significant differential expression in sepsis patients and are closely linked to the functional status of immune cells and clinical outcomes.

CD27, a member of the tumor necrosis factor receptor superfamily, is primarily expressed in T cells, B cells, and NK cells (33, 34). It acts as a costimulatory receptor that binds to the ligand CD70, recruiting signal-adapted molecules like TRAF2 and SHP-1. This interaction regulates the TCR and CD28 signaling pathways, promotes memory T-cell differentiation, and affects the intensity and duration of the inflammatory response (33, 34). In the early stages of sepsis, elevated CD27 expression may amplify the systemic inflammatory response by increasing pro-inflammatory cytokines such as IFN-γ. Conversely, in later stages, decreased CD27 expression may lead to an immunosuppressive phase, raising the risk of secondary infections (35–37). Thus, balancing CD27 expression is crucial for managing the inflammatory response in sepsis patients.

KLRB1 encodes the CD161 receptor, which is predominantly expressed on T cells and natural killer (NK) cells and participates in multiple immune processes (38). Genetic inactivation of KLRB1 or antibody-mediated blockade of CD161 enhances T-cell cytotoxicity against tumor cells (38). Although these findings derive from oncology, sepsis also features T-cell dysfunction, which suggests that KLRB1 could modulate the anti-pathogen immune response in sepsis through a comparable mechanism. Separately, SARS-CoV-2 infection has been reported to downregulate KLRB1 expression in NK cells and thus impair NK cytotoxicity (39); by extension, restoring KLRB1 expression might strengthen T/NK cell–mediated pathogen clearance. Clinical studies further link higher KLRB1 expression to better outcomes, including longer overall survival in sepsis patients (40). Consistent with those reports, our data show that elevated KLRB1 expression correlates positively with survival in sepsis.

RETN is primarily secreted by monocytes and is closely linked to inflammation and metabolic regulation (41–44). As a ligand, RETN engages receptors such as TLR4 and CAP1 and activates the NF-κB signaling pathway, which promotes release of large amounts of proinflammatory cytokines and drives the systemic inflammatory response in early sepsis (45, 46). In contrast, during the immunosuppressive phase of sepsis, RETN can display anti-inflammatory activity and protect the liver by modulating the Th17/Treg balance (45). In our cohort, high RETN expression correlated with poorer prognosis in sepsis patients, although its apparently stage-dependent, dual actions warrant further investigation.

CD163 is a hemoglobin clearance receptor prominently expressed on monocytes and macrophages, with especially high levels in M2-type macrophages (47). Primarily, CD163 functions to remove free hemoglobin and produce anti-inflammatory and antioxidant products by inducing HO-1 expression (48–50). During sepsis, changes in CD163 expression reveal the polarization state of macrophages. Its upregulation is closely linked to sepsis risk, as elevated expression in peripheral blood immune cells suggests patients may be in an immunosuppressive stage. Thus, CD163’s role is highly microenvironment-dependent, potentially offering both protective and pathological effects in sepsis (51).

Based on this study’s findings and existing research theories, we propose that CD27, KLRB1, RETN, and CD163 may collaboratively regulate immune homeostasis in sepsis through their interactions. CD27 and KLRB1 form an adaptive immune regulatory axis: CD27 potentially enhances KLRB1 expression by promoting T-cell activation and IFN-γ secretion, thereby modulating NK cell toxicity. The inhibitory signal mediated by KLRB1 may feedback regulate CD27’s continuous activation, preventing excessive immune responses (42, 52–54). Conversely, RETN and CD163 constitute the myeloid immune regulatory axis: RETN promotes the release of inflammatory factors via the TLR4/NF-κB pathway, potentially inhibiting CD163’s anti-inflammatory function. CD163 may, in turn, negatively feedback inhibit RETN expression by eliminating hemoglobin and its anti-inflammatory products (41, 55, 56). Notably, there is cross talk between these two immune axes: RETN can influence CD27 and KLRB1 expression by regulating monocyte function, whereas KLRB1+ T cells may affect macrophage polarization and CD163 expression by secreting cytokines such as IL-17 or GM-CSF (41, 56–58). This multidimensional regulatory network may initially present a pro-inflammatory state (dominated by CD27/RETN) in early sepsis, transitioning to immunosuppression (upregulation of KLRB1/CD163) in later stages. This dynamic balance may determine patients’ clinical outcomes.

In conclusion, our research revealed that CD27, KLRB1, RETN, and CD163 are highly specific and sensitive biomarkers for sepsis. Their functions hold significant potential for guiding sepsis treatment at various stages. Notably, the expression levels of KLRB1, RETN, and CD163 are closely linked to patient prognosis and may serve as potential targets for future sepsis immunotherapy.

## Data Availability

The datasets presented in this study can be found in online repositories. The names of the repository/repositories and accession number(s) can be found below: CNP0002611 (theChinaNationalGene Bank (CNGBdb, https://db.cngb.org/)).
